# Stretching Peptides’ Potential to Target Protein–Protein Interactions

**DOI:** 10.1021/acscentsci.3c00364

**Published:** 2023-04-12

**Authors:** Naresh M. Venneti, Jennifer L. Stockdill

**Affiliations:** Department of Chemistry, Wayne State University, Detroit, Michigan 48202, United States

Protein–protein interactions (PPIs) are key biological targets for next-generation drug discovery. Peptide-derived molecules are ideal for targeting PPIs because they can closely mimic the binding surfaces presented by the proteins in the PPI.^1^ Conformational control represents a major challenge in advancing peptides as PPI modulators for pharmaceutical development. In this issue of *ACS Central Science*, Philip Dawson and co-workers report an efficient approach to access “stretched” peptides, namely, those with an enforced geometry resembling that of a β-sheet.^2^ They accomplish this by installing a strained diyne-containing macrocycle and establishing the role of various modifications to the macrocycle’s structure in altering both the conformation of the peptide backbone and the antibiotic activity.

**Figure 1:**
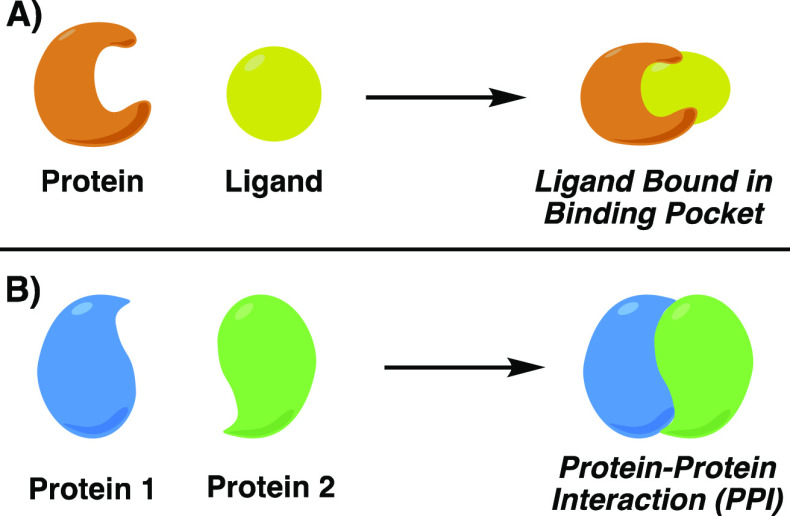
A) Traditional small-molecule drugging strategy targets clear binding sites. B) Broad surfaces of protein–protein interactions are difficult to target using small molecule ligands.

Historically, pharmaceutical agents have targeted interactions between proteins and their ligands, either through inhibition or augmentation of the protein’s function. The deep binding pockets involved in interactions of small molecules with proteins allow the design of highly active, selective small molecule inhibitors (Figure 1A). Targeting PPIs^1^ requires a major shift in design principles^3^ because of the comparably large, mostly flat nature of the surfaces involved. Without strong intermolecular interactions, overcoming the entropic costs of binding is difficult (Figure 1B). Meanwhile, the native proteins are evolutionarily well-matched to facilitate binding, and key “hot spots” in the binding surface—sites with deeper grooves or pockets—can be situated too far apart for a small molecule to efficiently capture multiple hot spot interactions. Furthermore, there are few natural products known to target PPIs, leaving chemists with a shortage of knowledge about critical interactions and limited inspiration for rational drug design.^4^

The discovery of natural products that interact with PPI interfaces could provide a critical advantage in interrupting PPI-mediated disease pathways. Indeed, natural products were represented either as isolated or in a modified form in ≥60% of new chemical entities approved by the FDA from 1981 to 2014.^5^ Most of the PPI-targeting candidate molecules currently in clinical trials are focused on cancer pathways.^6^ Because of the looming antibiotic resistance crisis, it is critical that new antibiotics be discovered and advanced to the clinic. Lead molecules with novel mechanisms of antibiotic activity are regarded as most likely to be able to defeat drug-resistant strains, and PPIs are emerging as promising biological targets for novel antibiotic development.^7^

**Figure 2:**
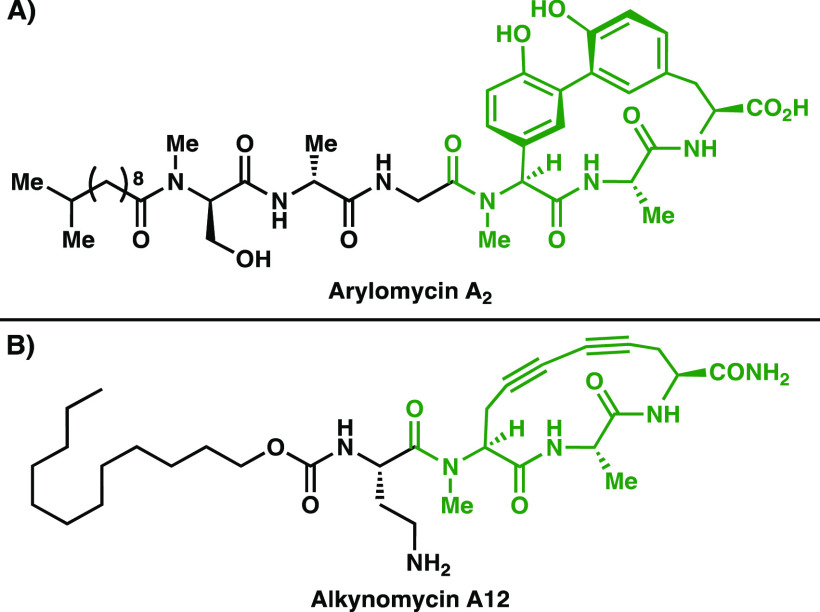
Representative (A) arylomycin and (B) alkynomycin structures. The macrocyclic portion including the critical H-bonding portion of the backbone is indicated in green. The diyne motif confers a similar conformational constraint as the biaryl motif.

The arylomycins (Figure 2A) are a promising family of lipopeptide antibiotics^8^ that target type I signal peptidase (SPase I), a conserved and essential bacterial enzyme, suggesting that bacteria are less likely to develop mutations causing resistance since mutation would likely result in a nonviable strain. Arylomycins and/or their derivatives have antibacterial activity in both Gram-negative and Gram-positive bacteria, and studies of their mechanism of action indicate that hydrogen bonding between the SPase surface and the arylomycin peptide backbone within the macrocyclic portion of the molecule is critical for binding.^9^ Additional requirements are the *N*-methylation of the N-terminal amide in the macrocycle and presence of the macrocyclic motif. However, X-ray analysis of arylomycin A_2_ bound to the surface of *Escherichia coli* SPase 1 indicates that the aryl groups are solvent-exposed—pointing away from the protein surface—and are likely not required for activity.^10^ Dawson and co-workers developed a series of constrained macrocyclic analogs bearing a diyne motif in place of the diaryl motif (Figure 2B). An important aspect of this research is the development of a fully on-resin approach to the alkynomycins, which makes rapid production of analogs a straightforward process. The efficiency of the on-resin oxidative Glaser coupling of two propargylalanine residues to form the constrained macrocycle is remarkable*.* This process requires bonding of the two alkynes and insertion of a transition metal into each of the electron-rich C_*sp*_–H bonds. This geometry would appear impossible to achieve by a single transition metal center, but the catalysis actually involves two copper atoms, making the catalytic intermediates energetically accessible. Furthermore, reactions performed on-resin are challenging to optimize and are often less efficient than their solution-phase counterparts. Dawson’s chemistry neatly addresses these challenges, and the on-resin approach avoids excessive solvent use caused by multiple purifications and renders the chemistry more user-friendly for the lay-chemist or biologist.

With multiple alkynomycins in hand, Dawson and co-workers systematically evaluated how changes to the size of the diyne macrocycle, the presence or absence of heteroatoms in the framework, and the α-stereochemistry affect the conformation of the backbone amides. They performed DFT calculations for each structure, noting the nonlinearity of the diyne motif, and then compared predicted geometries with NMR shifts and coupling constants. For a backbone amide in a β-sheet, coupling constants between 8 and 10 Hz are expected. The NMR data suggest that 13-membered ring diyne structures—where the diyne is bent a remarkable 40–50° out of linearity—most closely model a native β-sheet. Excitingly, these structures also exhibited the strongest binding activity to the surface of SPase 1, confirming the importance of the β-sheet conformation for binding to this surface. Analysis of the computed dihedral angles suggests that other peptide secondary structures and conformations could be accessed by fine-tuning the macrocyclic structure, either by substitution of heteroatoms and changes to the stereochemistry or via further reaction of the diyne motif to change the hybridization of the alkyne carbons. Additionally, this motif could be placed at either terminus or in the middle of a peptide sequence to further tune binding interactions. Thus, the readily accessible alkynomycin macrocyclic motif could prove to be a broadly useful design element for the discovery of novel PPI ligands.

With the power of structural biology and computational modeling, crucial information can be deduced about key interactions of a ligand with the protein surface.^1^ Combining conformational locks such as the alkynomycin macrocycle with structural biology, computational, and screening technologies will facilitate advancement of initial PPI-targeting molecules to the clinic.
